# Epigenetics in radiation biology: a new research frontier

**DOI:** 10.3389/fgene.2013.00040

**Published:** 2013-04-04

**Authors:** Matt Merrifield, Olga Kovalchuk

**Affiliations:** Department of Biological Sciences, University of LethbridgeLethbridge, AB, Canada

**Keywords:** radiation, epigenetics, DNA methylation, histones, small RNAs, genome instability, bystander effects, transgeneration effects

## Abstract

The number of people that receive exposure to ionizing radiation (IR) via occupational, diagnostic, or treatment-related modalities is progressively rising. It is now accepted that the negative consequences of radiation exposure are not isolated to exposed cells or individuals. Exposure to IR can induce genome instability in the germline, and is further associated with transgenerational genomic instability in the offspring of exposed males. The exact molecular mechanisms of transgenerational genome instability have yet to be elucidated, although there is support for it being an epigenetically induced phenomenon. This review is centered on the long-term biological effects associated with IR exposure, mainly focusing on the epigenetic mechanisms (DNA methylation and small RNAs) involved in the molecular etiology of IR-induced genome instability, bystander and transgenerational effects. Here, we present evidence that IR-mediated effects are maintained by epigenetic mechanisms, and demonstrate how a novel, male germline-specific, small RNA pathway is posited to play a major role in the epigenetic inheritance of genome instability.

## RADIATION CARCINOGENESIS

Life on earth has evolved in an environment subjected to ionizing radiation (IR). Humans are of no exception, as we are exposed to IR via the air we breathe, the food we eat, and from the sky above us (cosmic rays), as well as the ground we walk on. However, it is becoming increasingly common for humans to not only be exposed to natural or “background” levels of IR, but also to man-made sources of radiation. Owing to a marked increase in accessibility, and an introduction of innovative techniques that utilize IR, the number of people that receive chronic and/or acute exposures to radiation via occupational, diagnostic, or treatment-related modalities, is progressively rising. As civilization continues to progress, and radiation continues to be an integral part of modern life, the amount of man-made radiation will increasingly add to natural background exposure levels. Due in part to this, in recent years, much attention has been devoted to elucidate the biological responses and mechanisms underlying human exposure to IR.

Ionizing radiation is now universally accepted as a severe DNA damaging agent, which can lead to serious consequences, including cancer (Little, 1999). The majority of primary data on radiation-induced cancers in humans come predominantly from atomic bomb and nuclear accident survivors, as well as the medically exposed. A number of studies on survivors of the atomic bomb attacks on Japan demonstrated a greatly increased incidence of various cancers among survivors (Folley et al., 1952; Watanabe et al., 1972; Wakabayashi et al., 1983; Carmichael et al., 2003). Some of the largest accidentally exposed cohorts of people are currently available in the territory of the former Union of Soviet Socialist Republics (USSR), which include groups from major industrial accidents, such as the approximately 30,000 people who live near the Mayak nuclear facility in the southern Ural Mountains in Russia, the 1986 Chernobyl catastrophe, as well as from nuclear weapons testing in Kazakhstan (Dubrova, 2003a, b). The cohort of people in the Mayak region that were chronically exposed to IR demonstrated an increased incidence of leukemia, slightly lower than the rates experienced by atomic bomb survivors (Kossenko, 1996; Shilnikova et al., 2003). The 1986 Chernobyl accident led to a significant elevation in the rates of various cancers, including but not limited to, thyroid carcinoma, leukemia, and lymphoma (Gluzman et al., 2005; Balonov, 2007), breast cancer (Pukkala et al., 2006; Prysyazhnyuk et al., 2007), as well as bladder cancers (Romanenko et al., 2000; Morimura et al., 2004). In addition, elevated cancer and mutation rates were also reported in people living near the Semipalatinsk nuclear test site in Kazakhstan (Salomaa et al., 2002; Tanaka et al., 2006).

Even though IR is a well-known genotoxic agent and human carcinogen, it is also widely used to effectively diagnose and treat cancer (Little, 1999, 2000; Pollack et al., 2000; Roof et al., 2003; De Potter et al., 2006; Erven and Van Limbergen, 2007). Since 1902 when the first radiation-induced cancer was reported (Little, 2000, 2003), and almost 100 years after radiation was used for the first time to treat tumors (Gramegna, 1909), it still remains the number one diagnostic and treatment tool for the majority of cancers (Pollack et al., 2000; Roof et al., 2003; De Potter et al., 2006; Erven and Van Limbergen, 2007). While modern cancer radiation therapy has led to increased patient survival rates, the risk of treatment-related deleterious effects, including secondary cancers, is becoming a growing clinical problem (Leone et al., 1999). Relatively recent findings suggest that even fairly low doses of IR, such as those used in diagnostic procedures (e.g., X-ray or computer tomography), can lead to the development of radiation-induced cancers (Preston-Martin et al., 1989; Doody et al., 2000; Liu et al., 2002; Brenner and Hall, 2004). This risk of developing secondary treatment-related cancers is even more pronounced in children and young adults who received either diagnostic or therapeutic exposure to IR (Hildreth et al., 1989; Infante-Rivard et al., 2000; Hall, 2002; Shu et al., 2002; Kleinerman, 2006). In addition to children and young adults being more susceptible to secondary cancers, they also represent a special high-risk group for other possible delayed effects associated with IR exposure. Parental exposure to radiation from nuclear reprocessing plants, as well as through diagnostics, has been documented to result in a significant increase in the risk of leukemia and congenital malformations in their children (Shiono et al., 1980; Shu et al., 1988; Gardner et al., 1990; Nomura, 1993; Dickinson and Parker, 2002). Consequently, a major quality of life issue faced by young people who are exposed to radiation, especially young cancer patients and survivors, is not only an increased risk of secondary cancer development, but also the ability to produce healthy offspring. With an increasing number of individuals being exposed, it is becoming critically important to understand the full range of IR’s biological effects in order to properly assess and address the adverse impacts that it could have on humankind.

## CELLULAR EFFECTS OF DIRECT RADIATION EXPOSURE

Ionizing radiation has the ability to affect a variety of processes within exposed cells. It can cause changes in gene expression, disruption of mitochondrial processes, cell cycle arrest, and apoptotic cell death (Amundson and Fornace, 2003; Criswell et al., 2003; Fei and El-Deiry, 2003; Iliakis et al., 2003; Powell and Kachnic, 2003; Jeggo and Lobrich, 2006; Rodemann and Blaese, 2007; Valerie et al., 2007). IR’s ability to damage DNA by inducing a wide range of lesions is probably one of its most important and unique features affecting biological processes in mammalian cells (Frankenberg-Schwager, 1990). The lesions induced by IR include single- and double-strand breaks (SSBs and DSBs, respectively), as well as a varying complexity of DNA cross links and base damages. It has historically been accepted that incorrectly repaired DSBs are the principle lesion of importance regarding mutagenesis, and long-term biological effects associated with IR (Goodhead, 1994; Ward, 1995; Little, 2000). As a consequence of this damage not being repaired correctly, deleterious genetic changes such as mutations and chromosomal aberrations can be acquired at the initial sites of damage (Little, 2006). The accumulation of DNA damage caused by IR in conjunction with disrupted cellular regulation processes can lead to carcinogenesis (Little, 2000; Barcellos-Hoff, 2005; Sowa et al., 2006). To date, many studies have assessed the adverse impact of exposure to IR on human health in terms of mutation induction in somatic cells, using both *in vitro *and *in vivo *systems (Barber and Dubrova, 2006). As a result of these and other findings, it is now acknowledged that the adverse cellular effects and carcinogenic potential of radiation are not limited to what has historically been accepted. The historical assumption that the biological effects of radiation including cytotoxicity, mutation, and malignant transformations occur in the exposed cells themselves as a consequence of direct DNA damage may not always be the case. Biological effects associated with IR exposure can manifest in cells at delayed times after the initial insult, as well as in cells that did not receive direct irradiation (Morgan, 2003a, b). While the historical viewpoint data are still invaluable in providing information regarding health monitoring and risk assessment for directly exposed cells, they may significantly underestimate deleterious biological effects associated with IR exposure.

## DELAYED AND NON-TARGETED IR EFFECTS

### GENOMIC INSTABILITY

The significance of long-term or so-called “delayed effects,” associated with exposure to IR has become much more evident in recent years. It had long been thought that the main factor contributing to the negative biological effects of radiation in mammalian cells, such as chromosomal aberrations, mutations, and cell death, is the result of DNA damage in directly exposed cells; that is, residual damage that has not been repaired by the metabolic processes in the exposed cell (Little, 1998). This paradigm has largely been challenged in recent years, mostly from the results of numerous *in vitro *studies that demonstrated the existence of delayed effects associated with IR exposure (Morgan, 2003a). These delayed effects can manifest in the unexposed progeny of irradiated cells for many cell divisions (and up to 4 years) after the initial insult (Morgan, 2003a). The all-encompassing term given to this phenomenon is “radiation-induced genomic instability,” which is used to describe the increased rate of acquisition of alterations within the genome. Experimentally, genomic instability is observed when a cell is irradiated, then clonally expanded, and the progeny is examined genetically. As mentioned, radiation-induced genomic instability is observed generations after the initial exposure, and a number of studies have shown that this occurs at a high frequency (Limoli et al., 1999). Multiple genetic endpoints have been utilized to evaluate radiation-induced genomic instability in a number of *in vitro *systems, which include, but are not limited to, chromosomal aberrations, ploidy changes, micronucleus formation, gene mutations, amplifications, as well as increased microsatellite/expanded simple tandem repeat (ESTR) mutation rates and delayed cell death (Huang et al., 2003; Morgan, 2003a, b, c; Suzuki et al., 2003). There are a number of pathways implicated in the initiation and perpetuation of radiation-induced genomic instability (Kaplan et al., 1997). The relative amount of contribution of the different pathways primarily depends upon the genetic background of the irradiated cell or organism (Paquette and Little, 1994; Watson et al., 1997), as well as the type of radiation (Limoli et al., 2000).

A number of independent studies that have utilized *in vitro *systems have shown a high frequency of IR-induced genomic instability by means of examining the various endpoints (as described above) that are now associated with IR-induced genomic instability (Morgan, 2003a). Although there is still some speculation regarding the combined biological significance of these observations, the prevailing hypothesis is that IR exposure destabilizes the genome, thus initiating a cascade of genomic events that increases the rate of mutation and chromosomal change in the progeny of that irradiated cell (Morgan, 2003a). It has long been speculated that the development of genomic instability can facilitate the process of cancer initiation and/or progression (Cheng and Loeb, 1993), and indeed, the loss of genomic stability is believed to be a hallmark of many cancers, as well as an important prerequisite for cancer formation (Goldberg, 2003; Little, 2003; Loeb et al., 2003). Therefore, the general assumption is that there is a link between the induction of IR-induced genomic instability and cancer, due to an increase in the accumulation of multiple genetic events within a cell that ultimately enhance radiation-induced carcinogenesis. This assumption is also supported by the findings of epidemiological studies, which suggest that some types of radiation-induced cancers may follow a relative risk model, in which IR exposure enhances the rate at which cancers develop, instead of inducing a specific cohort of new tumors (Little, 2000). The demonstration of IR-induced genomic instability in somatic cell culture systems has greatly increased interest in research concerning the potential long-term effects for exposure. One such area that this has undoubtedly expanded to is the potential long-term effects associated with germline IR exposure and the transmission of adverse effects (e.g., genomic instability) to future generations.

### TRANSGENERATIONAL EFFECTS

The *in vitro *data, as mentioned above, have provided overwhelming evidence for delayed effects associated with IR exposure that can manifest in the progeny of irradiated cells (i.e., genomic instability) for many divisions, thereby enhancing the carcinogenic potential of these cells. Moreover, they point out that genomic instability could also be induced in the irradiated germline, and therefore may be transmitted to future generations. If this is the case, then the offspring of irradiated parents may be genetically unstable, resulting in a number of possible transgenerational effects, such as elevated mutations rates and a predisposition to cancer. Many publications have indeed characterized a wide variety of phenotypic traits observed in the offspring of irradiated parents, implicating increased mutation rates. Such studies have been reinforced through the use of various molecular techniques used to assess transgenerational genomic instability. Here, we will briefly outline some of the main “classical” publications that have mainly analyzed hereditary phenotypic alterations associated with parental exposure. This will be followed by the chief molecular and genetic studies/techniques that have backed these finding by demonstrating genomic instability in the progeny of irradiated parents (i.e., transgenerational genomic instability).

The first evidence for a transgenerational effect associated with IR exposure was demonstrated by Luning et al. (1976), where elevated rates of dominant lethal mutations (early and late embryonic death) were observed following the intraperitonial injection of male mice with a plutonium salt solution. Accordingly, an increase in dominant lethality not only occurred from the germ line of directly irradiated male mice, but also from the germline of their non-exposed, first-generation mice (F1; Luning et al., 1976). The offspring of irradiated male mice have also been shown to be reproductively challenged, exhibiting decreased fertilization rates for both *in vivo *and *in vitro *fertilization (Lyon, 1964; Burruel et al., 1997), as well as increased levels of prenatal mortality for the F2 generations (Pils et al., 1999). An increase in teratogenic effects was also shown, as the number of malformed F2 fetuses was significantly higher in the paternally exposed group compared to the control (Pils et al., 1999). Nomura (1982, 2003), not only demonstrated that paternal irradiation leads to an increase in malformations in the progeny of irradiated parents, but also to a significant increase in the incidence of cancer among these offspring. Several additional transgenerational studies also found a significant increase in cancer incidence among the offspring of paternally irradiated mice following secondary exposure to known carcinogens (Nomura, 1982; Lord et al., 1998; Hoyes et al., 2001). The predisposition of the offspring of IR-exposed fathers to cancer has also been investigated in human populations, where the data obtained have mainly been inconclusive (Roman et al., 1999; McKinney et al., 2003); however, two independent studies have shown a clustering of extremely high leukemia rates in children whose fathers had been exposed to radiation after working at a nuclear processing plant in the town of Sullafield (Gardner et al., 1990; Dickinson and Parker, 2002).

Adding to the classical evidence of transgenerational impacts, the majority of recent data have arisen from various molecular techniques used to characterize genotypic alterations in unexposed offspring. Mainly, the genotypic alterations found in the progeny of irradiated parents have included chromosomal aberrations, micro nuclei formation, increased minisatellite/ESTR mutations, and altered gene expression patterns, which are all hallmarks of genomic instability (Dubrova, 2003a, b, c; Morgan, 2003a; Barber and Dubrova, 2006). The manifestation of such alterations has, therefore, collectively been termed transgenerational genome instability. Dubrova (2003b, c) have made a significant contribution to the current understanding of radiation-induced transgenerational genome instability by pioneering the investigation of transgenerational mutation rates within repetitive sequences of the genome. These repetitive sequences were initially termed minisatellites, but are now known as ESTR loci, because they are long homologous arrays of relatively short (4–6 bp) repeats that show high spontaneous mutation rates in germline and somatic cells, whereas true minisatellites generally consist of longer (10–60 bp) repeats with much lower somatic mutation rates (Kelly et al., 1989; Gibbs et al., 1993; Bois et al., 1998; Dubrova, 2003b). Barber and colleagues studied mutation rates of two ESTR loci in the germline of F1 and F2 offspring of male mice exposed at either the pre-meiotic or post-meiotic stages of spermatogenesis (Barber et al., 2002). They found an increased mutation rate in the germline of F1 offspring, which was similarly maintained in the germline of the F2 offspring, for both pre/post-meiotic germ cell exposure groups. Furthermore, the elevated mutation rates were seen in all three of the mice strains they studied, and within each strain, male and female offspring (both F1 and F2) of irradiated fathers equally demonstrated elevated mutation rates (Barber et al., 2002). Further analysis of the unexposed F1 progeny showed that high ESTR mutation rates were observed along with elevated mutations in protein-coding genes in germline, as well as in somatic tissues, such as spleen and bone marrow (Barber et al., 2006). The analysis of mutation rates in genomic repeat elements has also been applied to study transgenerational IR effects in human populations, namely in individuals living in the vicinity of the Chernobyl reactor accident or near nuclear test sites (Semipalatinsk, Kazakhstan; Dubrova et al., 1996, 2002a, b). In all of these studies, they found an increase in the mutation rate among the progeny of the exposed parents. Taken together, these data support the hypothesis that exposure to IR can induce germline genomic instability that may predispose future generations to an increase risk of genetic diseases, infertility, and even cancer.

Similarly to IR, a variety of genotoxic agents were shown to induce germline and transgenerational effects in rodent models (Robison and Mertens, 1993; Nomura, 2006; Anway et al., 2008; Dubrova et al., 2008; Nomura, 2008). Also, there is strong evidence that paternal exposure to anticancer drugs can cause heritable genetic damage and diseases in their offspring (Robison and Mertens, 1993; Shelby, 1996; Witt and Bishop, 1996). Importantly, exposure to clinically relevant doses of bleomycin, cyclophosphamide, and mitomycin C led to statistically significant, dose-dependent increases in mutation frequencies in the germline of treated male mice (Glen et al., 2008). Moreover, particulate air pollution was also shown to affect the male germline and lead to DNA damage, germline mutation and altered global DNA methylation in murine sperm (Yauk et al., 2008).

### BYSTANDER EFFECTS

In addition to genome instability and transgenerational effects, the paradigm of genetic alterations being restricted to directly hit cells has also been challenged by numerous observations in which cells that were not directly traversed by IR, but were either in the neighborhood of irradiated cells or exposed to factors produced by irradiated cells, exhibited responses similar to those of the directly exposed cells (Morgan, 2003a, b; Morgan and Sowa, 2005). Such “non-targeted” effects are collectively regarded as radiation-induced “bystander effects”; accordingly, naïve cells exhibiting these responses are commonly called “bystander cells.” 

Evidence supporting the phenomenon of the bystander effect has been demonstrated in studies performed as early as the beginning of the 20th century. Murphy and Morton, whose research interests were devoted to the study of lymphoid cells, showed morphological changes in lymphoid cells after culturing them with serum from radiation-exposed animals (Murphy and Morton, 1915; Murphy et al., 1922). Additionally, Parsons et al. (1954) reported the presence of soluble clastogenic factors in the circulating blood of patients who underwent radiotherapy. Clastogenic factors are known for their ability to induce chromosome damage in cultured cells (Goh and Sumner, 1968; Hollowell and Littlefield, 1968; Emerit et al., 1994, 1995). Such clastogenic activity has also been demonstrated in the plasma from patients who received high dose radiotherapy, and from individuals accidentally exposed to radiation from the Chernobyl accident (Goh and Sumner, 1968; Pant and Kamada, 1977; Emerit et al., 1994, 1995); however, the term bystander effect was in fact not coined until the 1990s, when it was adopted from the gene therapy literature, where it was used to describe the killing of several tumor cell types after targeting only one type of cell within a heterogeneous population (Freeman et al., 1993). Direct studies of bystander effects have most widely been done *in vitro, *and the most common experimental model used to study it has generally involved the exposure of monolayer cultures to very low fluencies of α-particles, such that only a small fraction of the total cell population is hit by a particle (Nagasawa and Little, 1992; Little, 2000). In the initial report of this phenomenon, an enhanced frequency of sister chromatid exchanges (SCEs) was observed in up to 50% of the cell population, when only 0.1–1% had been traversed by radiation (Nagasawa and Little, 1992). The authors noted that frequency of SCE significantly increased with increasing exposure time and further reached a plateau at 2.45 mGy. At the plateau, the frequency of SCE was about 1.4 times the background level (Nagasawa and Little, 1992). In the late 1990s, there was resurgence in the interest and awareness of radiation-induced bystander effects, due largely to the development of charged-particle microbeam irradiators (Folkard et al., 1997). The microbeam is capable of putting an exact number of particles through specific subcellular compartments of a defined number of cells in a particular radiation environment (Folkard et al., 1997; Randers-Pehrson et al., 2001). The most convincing demonstration of the bystander effect has employed this technique, demonstrating that not only nuclear, but even cytoplasmic irradiation can have genetic consequences, both of which can be manifested in bystander cells (Wu et al., 1999).

Since then, a variety of cell culture studies have, indeed, demonstrated radiation-induced bystander effects with different endpoints being observed depending on the type of cells receiving/producing the bystander signal, as well as the type of radiation (Lorimore et al., 2003; Morgan, 2003a). Some, but not all, of these endpoints are detrimental to the cell. Similar to genomic instability, bystander effects are measured by the induction of gross chromosomal rearrangements, chromosome aberrations, SCEs, deletions, duplications, mutations, amplifications, and cell death (Kovalchuk and Baulch, 2008). Bystander effects such as these have also been demonstrated in 3D tissue models (Persaud et al., 2005), and in reconstructed human tissue models (Belyakov et al., 2005; Sedelnikova et al., 2007). As a result, bystander effects are accepted as a ubiquitous consequence of radiation exposure (Mothersill and Seymour, 2004). By the nature of their occurrence bystander effects can be grouped into two separate, but not necessarily mutually exclusive, mechanisms for the transfer of a signal from irradiated cells to naïve cells. One mechanism of the bystander effect is gap-junction communication-mediated, and is based on the ability of intercellular gap junctions to transmit some type of signal from irradiated to non-irradiated cells (Bishayee et al., 2001; Azzam et al., 2003a, b; Shao et al., 2003; Suzuki and Tsuruoka, 2004). The other proposed mechanism is known as a medium-mediated bystander effect, and is based on the ability of irradiated cells to secrete certain factors into the medium that are then received by non-irradiated cells (Lyng et al., 2002; Zhou et al., 2002; Yang et al., 2005a; Liu et al., 2006; Maguire et al., 2007). Although candidate signaling molecules are numerous, current literature suggests key players include reactive oxygen species (ROS; Lyng et al., 2000, 2002; Azzam et al., 2003b; Mothersill and Seymour, 2004), cytokines (Iyer et al., 2000; Facoetti et al., 2006), Ca^2^^+^ ions (Lyng et al., 2000, 2002, 2006), and notably short RNA (Koturbash et al., 2007; Kovalchuk and Baulch, 2008; Ilnytskyy and Kovalchuk, 2011). Thus far, examinations of bystander effects *in vivo* have been relatively scarce, nevertheless, when extrapolated to organisms as a whole, the results from cell and tissue culture experiments suggest several key possibilities: (1) communication of bystander signals through cell-to-cell gap junctions means that more cells are affected by a single localized exposure than predicted by the current target model for low dose exposure; (2) media transfer experiments suggest that exposed cells are able to secrete some type of signaling molecule or factors into the bloodstream and cause bystander effects anywhere in the body; (3) the extent to which bystander effects are manifested, and genomic instability is induced may largely depend on the type of tissue, and genetic background of organism.

As previously mentioned, the occurrence of bystander effects *in vivo *had long been suggested ever since it was shown that exposure to radiation produces “clastogenic” factors in the circulating blood of exposed animals and humans (Murphy and Morton, 1915; Murphy et al., 1922; Parsons et al., 1954; Emerit et al., 1994). However, compared to data from cell culture studies the conclusive data involving the molecular etiology of IR-induced bystander effects *in vivo, *especially those concerning the germ line, are rather sparse (Goldberg and Lehnert, 2002; Hall, 2003; Koturbash et al., 2006b, 2007; Mothersill et al., 2007; Tamminga et al., 2008b). Nevertheless, IR-induced bystander effects have been confirmed to occur within the exposed organs. A study utilizing an animal model was able to show that when only the base of the lung was irradiated significant molecular and cellular damage occurred in the shielded lung apex (Khan et al., 1998, 2003). It was also shown that when one lung was exposed there was a marked increase of micronuclei in the other unexposed/shielded lung (Khan et al., 1998, 2003). Similar intra-organ bystander effects were observed in a rodent model that underwent partial liver irradiation (Brooks et al., 1974; Brooks, 2004). Not surprisingly, it has more recently been shown that bystander effects also manifest themselves in the context of an organism in its entirety. In order to analyze the role of epigenetic changes associated with radiation-induced bystander effects *in vivo*, Kovalchuk and colleagues used a murine model system whereby half of an animal’s body was exposed to radiation, while the other half was protected by a medical grade lead shield (Koturbash et al., 2006b). They confirmed the existence of somatic bystander effects, by showing that X-ray exposure to one side of an animal’s body caused profound epigenetic changes in the unexposed bystander portion of the animal’s body (Koturbash et al., 2006b, 2007). In these studies they also found that male mice exhibited a more pronounced bystander effect. It has recently been shown for the first time that localized cranial exposure causes an *in vivo *bystander response, not only in somatic tissue but in the male germline as well (Tamminga et al., 2008a). In addition, it was shown that bystander damage to the germline caused by localized cranial irradiation had transgenerational consequences, causing profound epigenetic alterations in the unexposed progeny (Tamminga et al., 2008b).

## EPIGENETICS OF IR EXPOSURE 

A plethora of information available in the literature from *in vitro *studies, as well as compelling data from whole organisms, has provided convincing evidence for the existence of IR-induced bystander, as well as transgenerational effects, both of which have been linked to the phenomenon of IR-induced genomic instability. Notwithstanding are the underlying molecular mechanisms that lead to their development; however, there is strong evidence for a common underlying molecular mechanism linking these phenomena. This is most compellingly evident in the commonality of the end points observed for these phenomena (i.e., of genomic instability). A high frequency of induction and persistence of IR-induced genomic instability, as well as a non-Mendelian mode of inheritance of transgenerational effects suggests an epigenetic based mechanism (Wiley et al., 1997; Lorimore et al., 2003; Morgan, 2003a, b; Nagar et al., 2003; Barber et al., 2006; Kaup et al., 2006; Wright and Coates, 2006; Kovalchuk and Baulch, 2008) 

Epigenetic alterations are meiotically heritable and mitotically stable alterations in gene expression with no change in DNA sequence, which include DNA methylation, histone modifications, and RNA-associated silencing (Jaenisch and Bird, 2003).

### DNA METHYLATION

DNA methylation was the first epigenetic alteration identified, and is the most widely studied epigenetic mechanism. In mammals, DNA is methylated at the carbon 5 of cytosine residues to form 5-methyl-cytosines (5meC), which is established by the *de novo *DNA methyltransferases (DNMT3a, DNMT3b, and DNMT3L), and subsequently maintained by DNMT1 (Robertson, 2001; Rountree et al., 2001; Goll and Bestor, 2005). The *de novo *DNA methylation of transposons in the germline is dependent on DNMT3L, an isoform of DNMT3a and DNMT3b that lacks methylation activity (Kato et al., 2007). DNA methylation is known to be associated with inactive chromatin states, and in most cases, with the repression of gene expression (Hendrich and Tweedie, 2003; Klose and Bird, 2006; Weber and Schubeler, 2007). Proper regulation of DNA methylation is critically important for normal development, cell proliferation, and the maintenance of genomic stability within a given organism (Ehrlich, 2002; Robertson, 2002; Jaenisch and Bird, 2003). The global loss of DNA methylation has been linked to the activation of transposable elements (TEs), elevated chromosome breakage, aneuploidy, increased mutation rates, and therefore to the phenomenon of genomic instability (Robertson, 2002; Weber and Schubeler, 2007; Weidman et al., 2007). In addition, altered global DNA methylation patterns are a well-known characteristic of cancer cells, and global loss of cytosine methylation was the first epigenetic abnormality discovered in cancer cells (Feinberg and Vogelstein, 1983; Flatau et al., 1983; Gama-Sosa et al., 1983; Feinberg, 2004). The DNA methylation profile of cancer cells is frequently characterized by global genome hypomethylation, as well as concurrent hypermethylation of selected CpG islands within gene promoters (e.g., tumor suppressor; Jaenisch and Bird, 2003; Baylin, 2005; Baylin and Ohm, 2006; Weidman et al., 2007).

Consequently, it is not surprising that direct IR exposure has been reported to affect DNA methylation patterns. Acute exposures to low linear energy transfer (LET) radiation such as X-rays and/or γ-rays have been noted to result in global genomic DNA hypomethylation (Weidman et al., 2007). More recently, IR exposure has been found to lead to profound dose-dependent, as well as sex and tissue-specific global hypomethylation (Pogribny et al., 2004; Raiche et al., 2004; Koturbash et al., 2005; Loree et al., 2006). This loss of methylation was also associated with radiation-induced alterations in the expression of DNA methyltransferases, notably *de novo *methyltransferases DNMT3a and DNMT3b (Raiche et al., 2004; Pogribny et al., 2005). Most importantly, the radiation-induced global DNA hypomethylation patterns appear to be linked to genomic instability in the exposed animals (Pogribny et al., 2004, 2005; Raiche et al., 2004; Loree et al., 2006).

DNA methylation also plays a role in radiation-induced bystander effects. Kaup et al. (2006) lead the way in showing the importance of DNA methylation in the maintenance of radiation-induced bystander effects. They demonstrated that dysregulation of DNA methylation patterns occurs in non-irradiated cells and can persists for 20 passages when they are treated with medium from irradiated cells (Kaup et al., 2006). These bystander cells, marked with aberrant methylation patterns, also exhibited numerous endpoints characteristic of genome instability (Kaup et al., 2006). The same pattern of genomic instability and significant loss of nuclear DNA methylation was also observed in 3D human tissue models (Sedelnikova et al., 2007). Some of the first data to clearly demonstrate that epigenetically regulated bystander effects occur *in vivo *came from a murine model study which showed that radiation exposure, leads to elevated levels of DNA strand breaks, and altered levels of key proteins involved in establishing and maintaining methylation marks, in lead shielded tissue at least 0.7 cm from irradiated tissue (Koturbash et al., 2006b). Using localized cranial X-ray irradiation on a rat model, Koturbash et al. (2007) also demonstrated that localized IR exposure can induce profound global DNA hypomethylation in distant bystander tissue (spleen), that was observed 24 h after exposure. Importantly, these changes were still observed seven months after exposure (Koturbash et al., 2007). This is significant in terms of carcinogenesis due to the fact that the epigenetic manifestations of bystander effects persisted over a long period of time, roughly equivalent to 10 years in humans. Again, the profound and persistent reduction of methylation in the bystander spleen was paralleled by altered (decreased) levels of key proteins involved in the establishment and maintenance of methylation patterns (i.e., DNMT3a, DNMT1, and methyl-binding protein MeCP2; Koturbash et al., 2007). This was believed to contribute to the observed reactivation of the long interspersed element-1 (LINE-1) retrotransposon in the bystander spleen (Koturbash et al., 2007). This experimentally observed hypomethylation was also shown to manifest in the bystander germline of cranially exposed rats. As such, methylation levels of LINE-1 retrotransposon were 2.2 times lower in the germline of bystander-exposed rats as compared to control animals (Tamminga et al., 2008b).

Consequently, the involvement of the same type of epigenetic effectors (DNA methylation, and associated proteins), in transgenerational effects induced from the paternal whole body, as well as localized cranial exposure to IR, have also been studied (Koturbash et al., 2006a; Tamminga et al., 2008b; Filkowski et al., 2010). Paternal whole body and cranially localized IR-exposures were shown to result in a significant 1.4, 1.55, and 1.4 times global loss of DNA methylation in the thymus, bone marrow, and the spleen of F1 offspring, respectively, as compared to the control offspring (Koturbash et al., 2006a; Tamminga et al., 2008b). Cranial exposure also resulted in specific hypomethylation of LINE-1 and short interspersed nuclear element (SINE) B2 in the germline of exposed males, which was further observed in the thymus of unexposed offspring. In the offspring, paternal cranial irradiation let to a significant 3.8 and 2.2 tiles decreased in methylation of LINE-1 and SINE B2, respectively (Tamminga et al., 2008b). Correspondingly, the thymus from the progeny of paternal whole body IR exposures, and bone marrow from the offspring of cranial exposed fathers, where the most pronounced decreases in DNA methylation was observed, also exhibited significant decreases in the expression of DNMT1, DNMT3a, DNMT3b, and methyl-binding protein MeCP2 (Koturbash et al., 2006a; Tamminga et al., 2008b). The global loss of DNA methylation and altered levels of methyltransferases and methyl-binding proteins can lead to the activation of TEs, contributing to genomic instability (Xu et al., 1999; Yu et al., 2001; Jirtle and Skinner, 2007). Accordingly, it may also be suggested that the global/site-specific loss of DNA methylation observed in the progeny of irradiated fathers may influence retrotransposons and satellite DNA, thus underlying transgenerational genome instability. Such a hypothesis also corroborates, and may help elucidate, the increased mutation rates in satellite DNA and ESTR loci observed in the progeny of exposed parents (Barber and Dubrova, 2006). Even though these epigenetic alterations are well-characterized consequences of radiation exposure, the underlying molecular mechanism that drive these alterations, especially site-specific changes in DNA methylation patterns, remain elusive. Such molecular mechanisms may very well be chief contributors to IR-induced epigenetic alterations associated with germline genomic instability, and therefore, would be strongly implicated in facilitating epigenetic inheritance of transgenerational IR effects.

### HISTONE MODIFICATIONS

Indeed, changes in DNA methylation do not occur as isolated events, as they are closely connected to other components of chromatin structure, such as histone modifications (Jaenisch and Bird, 2003; Weidman et al., 2007). The main histone modifications include acetylation, methylation, phosphorylation, and ubiquitination (Jenuwein and Allis, 2001). There is a vast complexity of epigenetic control that can be exhibited from such modifications, as each of these modifications all have differing transcriptional consequences compounded by further control depending on which residue is modified, and to what extent (e.g., mono-, di-, tri-methylated; Cheung and Lau, 2005; Saha et al., 2006; Weidman et al., 2007; He et al., 2008). Histone modifications and DNA methylation closely interact in the setting of the transcriptional states of chromatin. Combinations of different histone modifications and other chromatin-binding proteins define the structural and functional status of chromatin.

One of the best studied histone modifications, especially regarding IR exposure, is the phosphorylation of histone H2AX at serine 139 (γH2AX). γH2AX is possibly one of the earliest cellular responses to DSB, and therefore, to IR exposure. The formation of γH2AX is crucial for the repair of DSB and for the maintenance of genome stability (Rogakou et al., 1998; Pilch et al., 2003; Sedelnikova et al., 2003). The involvement of H2AX phosphorylation in bystander, as well as transgenerational IR effects, has also been suggested. Elevated levels of γH2AX have been reported in somatic and notably germline bystander tissues *in vivo*, and this elevation has subsequently been observed in the offspring of exposed fathers (Barber et al., 2006; Koturbash et al., 2006a, b, 2007; Tamminga et al., 2008b).

Recent studies have indicated that IR-induced global loss of DNA methylation can be associated with changes in histone methylation patterns, specifically with the loss of histone H4 lysine tri-methylation (Pogribny et al., 2005). It was shown that human breast tumors have loss of tri-methylation at lysine 20 on H4 histones, accompanied with DNA hypomethylation, which has been suggested as a universal marker for malignant transformation (Sanders et al., 2004; Fraga et al., 2005; Tryndyak et al., 2006).

### SMALL RNA MEDIATED EVENTS

Epigenetic control can also be regulated by small RNA mediated events (Bernstein and Allis, 2005). Here, we will discuss two types of small regulatory RNAs that are of particular interest: microRNAs (miRNA) and Piwi-interacting RNAs (piRNAs). miRNAs are abundant small (~21–25 nt) single-stranded non-coding RNAs that regulate gene expression primarily at the post-transcriptional level (e.g., post-transcriptional gene silencing, PTGS). Initially, miRNAs are endogenously transcribed as part of a primary transcript (pri-miRNA) that is able to form one or more hairpin structures (miRNA stem loops) from complementary sequences within the transcript. MiRNA genes can be transcribed independently, or clustered with others and transcribed as a polycistron (Chen and Meister, 2005). There are also a large number of intragenic miRNAs transcribed from within introns or exons of protein-coding and non-coding genes (Rodriguez et al., 2004). These primary transcripts are then processed in the nucleus into stem-loop-structured miRNA precursors (pre-miRNA) approximately 70 nt long, by the RNase III enzyme Drosha. They are then exported to the cytoplasm where Dicer (RNase III enzyme) generates characteristic 21–25 nt long dsRNA that separate into two strands, one of which is incorporated into a member of the Argonaute protein family (AGO2), a central component the miRNA ribonucleoprotein complex (miRNP), commonly known as the RNA-induced silencing complex (RISC; Zeng, 2006). To control the translation of specific mRNAs, the miRNA guided RISC complex binds to the 3′untranslated region (UTR) of target mRNAs with a similar sequence structure, thus serving as translational repressors that regulate protein synthesis by targeting specific mRNAs (Hutvagner and Zamore, 2002). Currently, it is believed that miRNAs exhibiting a high degree of complementarity to their target mRNAs are able to repress translation through mRNA cleavage. However, most miRNAs in mammals have imperfections between the complementary sequences, and therefore, repress translation without cleavage (Doench and Sharp, 2004; Yekta et al., 2004). Although the precise nature of such regulation remains unclear, it is suggested that the main mechanisms include alteration of pol(A) tail length and binding of regulatory proteins to the UTRs of target mRNAs (Grivna et al., 2006). One or many miRNAs can coordinate the expression of single/multiple genes, resulting in a complex mechanism for post-transcriptional gene regulation. Consequently, miRNAs can play key roles in numerous biological contexts, including cellular differentiation, proliferation, apoptosis, and even a predisposition to cancer (Shivdasani, 2006; Chang and Mendell, 2007; Fabbri et al., 2007). In addition, altered levels of miRNAs have been reported in a variety of cancers (Volinia et al., 2006; Wiemer, 2007). A number of miRNAs are deleted or silenced in cancer and therefore identified as onco-suppressors, while others, inversely, are overexpressed and considered oncogenic (Hwang and Mendell, 2006; Aqeilan et al., 2010).

Not unexpectedly, miRNAs are also involved in IR-induced responses *in vivo*. IR exposure to one half of a mouse’s body triggered a significant upregulation of *miR-194 *in distant bystander liver tissue, which was suggested to initiate and maintain the observed downregulation of DNMT3a and MeCP2 in the same bystander tissue (Koturbash et al., 2007). Altered expression patterns of miRNAs have also been profiled in directly exposed males, as well as their unexposed offspring, demonstrating the possibility that they may also play a role in transgenerational epigenetic inheritance of genomic instability (Filkowski et al., 2010). It was found that paternal irradiation lead to an upregulation of the miR-29 family in the exposed male germline, which was believed to cause decreased expression of* de novo* methyltransferase DNMT3a, and profound hypomethylation of LINE-1 and SINE B2 (Filkowski et al., 2010). Furthermore it was also shown that paternal irradiation caused a significant upregulation of miR-468 in thymus of progeny, causing decreased expression of a lymphoid-specific helicase (LSH) crucial for the maintenance of methylation and silencing of repetitive elements (Filkowski et al., 2010).

**FIGURE 1:**
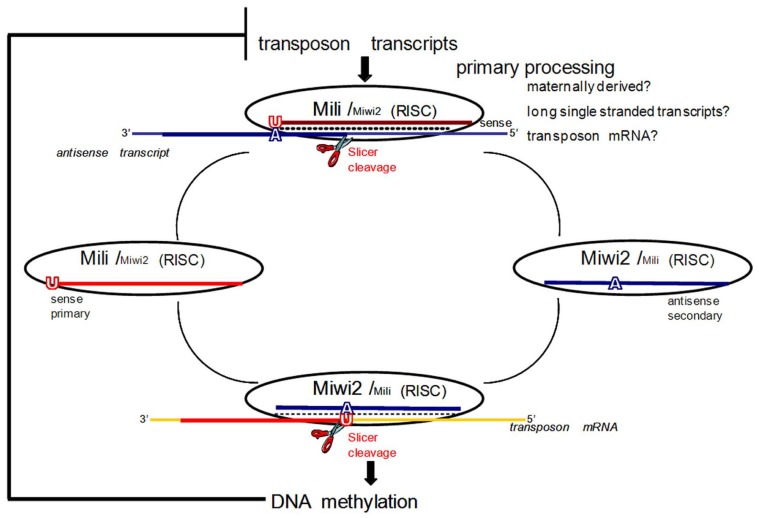
**The ping-pong model for piRNA amplification in mice.** In mice primary processing results in sense piRNAs that preferentially associate with MILI. In prenatal testis both MILI and MIWI2 participate in the amplification cycle. MIWI2 is specifically enriched in secondary antisense piRNAs as compared to MILI. Antisense secondary piRNAs guide DNA methylation of transposable element sequences. After birth, when MIWI2 is no longer expressed, MILI is believed to continue to operate in the cycle alone. If DNA methylation of transposon sequences is impaired due to downstream mutations in methyltransferase proteins, overexpression of transposon transcripts boosts primary processing and increases the fraction of primary sense piRNAs. Adapted with permission from Aravin et al. (2008).

Recently, an additional novel small RNA pathway has begun to be characterized, providing evidence for yet another small RNA mediated epigenetic effector. Known as the Piwi/piRNA pathway, it has several unique features that make it quite suitable as a mediator of epigenetic memory in germ cells. Initially characterized in *Drosophila* (Aravin et al., 2003), the central components of the pathway are a large class of short, single-stranded, non-coding RNAs (~26–31 nt) and their Piwi protein partners, a subclass of the Argonaute protein family. Both piRNAs and Piwi proteins have expression patterns that are largely restricted to germ cells in nearly all multicellular animals studied (Aravin and Hannon, 2008). Piwi proteins are required for the production of their piRNA partners, and are essential for various stages of spermatogenesis, as well as germ stem cell self-renewal and transposon silencing (Aravin and Hannon, 2008; Thomson and Lin, 2009). The best studied function of the piRNA pathway is to maintain genomic integrity by the suppression of TEs, via transcriptional gene silencing (TGS; Aravin and Hannon, 2008). TGS occurs through piRNA pathway mediated *de novo *methylation of the regulatory regions of retrotransposons in embryonic germ cells, which is believed to be subsequently maintained in germ and somatic cells throughout the life of the organism (Aravin and Hannon, 2008; Kuramochi-Miyagawa et al., 2008). While mutations in the DNA (cytosine-5-)-methyltransferase (DNMT) family members impacted cytosine methylation, the piRNA pathway remained largely unaffected (Aravin and Hannon, 2008). In contrast, a loss of the piRNA pathway prevents recognition and silencing of TE by DNMT3L, supporting a model in which the piRNA pathway acts upstream of DNMT3L, and consequently DNMT3a and DNMT3b, to establish patterns of DNA methylation on TEs (Aravin and Hannon, 2008). PTGS also contributes during this process as piRNA guided Piwi proteins, indicative of the miRNA RISC complex, mediate cleavage of active transposon mRNA, from which primary piRNAs are believed to be derived in a process known as the “ping-pong” amplification cycle (Aravin et al., 2007a, b; **Figure 1**). However, it is important to note that the majority of mouse and rat piRNAs are not enriched for sequences from transposons and repeats. In mice and rats, repeats are underrepresented, since only ~17% of all piRNAs map to repetitive elements while a random distribution should yield close to 40%, which is the proportion of repetitive sequences in the genome (Vagin et al., 2006; Hartig et al., 2007). In mammals, piRNAs tend to cluster within certain regions of the genome, and a large number of piRNAs are derived from intergenic regions, but are also distributed among exonic, intronic, and as mentioned, repeat sequences (Grivna et al., 2006). A distinguishing feature of these clusters of uniquely mapping piRNAs is their pronounced strand bias, thereby leading to the proposal that the biogenesis of piRNAs involves a long, single-stranded precursor (Seto et al., 2007). Given that piRNA sequences correspond to a variety of genomic regions, the piRNA pathway may be involved in a more complex system, regulating the expression of genes other than repetitive elements.

Indeed, several recent studies suggest that the piRNA pathway is not limited to the repression of transposable and repetitive elements, and may have additional diverse and complex roles in regulating gene expression at all known levels of epigenetic control. Piwi proteins and piRNAs together have been associated with mRNA, and mRNA cap-binding proteins in polysomes and ribonucleoproteins (RNPs), which play central roles in translational control; however, the molecular mechanisms that achieve this translational regulation and the resulting outcomes remain unclear (Grivna et al., 2006; Thomson and Lin, 2009; Unhavaithaya et al., 2009). A biochemically purified endogenous rat piRNA complex has been shown to exhibit RNA cleavage activity, presumably facilitated by the rat Piwi protein, Riwi (Lau et al., 2006). On the other hand, mouse Piwi proteins may actually be responsible for the stability of a subset of mRNAs, and positively regulating translation (Deng and Lin, 2002; Unhavaithaya et al., 2009). In addition, Grivna et al. (2006) showed that a Piwi protein in mice (Miwi) is not only required for piRNA production, but also for a particular subset of miRNAs. Thus, the piRNA pathway may also be involved in miRNA-mediated translational control. One common feature of *Piwi *gene mutations in mice is an increase in DNA damage marked by γH2AX foci, suggesting a possible link to DNA damage repair/checkpoints (Kuramochi-Miyagawa et al., 2004; Carmell et al., 2007). It has been proposed that such dsDNA breaks are a result of over active transposons; however, this relationship is not fully understood, as dsDNA breaks could also be the cause of transposon activity, and not necessarily a result of it (Klattenhoff et al., 2007). Consistent with a possible role of mammalian Piwi-type proteins in DNA repair processes is the presence of RecQ1 in rat Piwi protein complexes (Lau et al., 2006). RecQ is a family of helicase enzymes that have highly conserved roles in dsDNA break repair through recombination (Hunter, 2008). The ability of the piRNA pathway to mediate epigenetic control of gene expression on the level of histone modifications has also been described. Human cells were transiently transfected with a human Piwi (Piwi-like4/Hiwi2) gene containing a vector construct, which induced histone H3K9 methylation at the p16Ink41 locus, resulting in significant downregulation of p16 gene expression (Sugimoto et al., 2007). A more recent study has provided intriguing evidence for the production and function of a particular subset of abundant piRNAs, which are depleted in TE content and do not engage in the ping-pong cycle (Robine et al., 2009). They reported a substantial population of piRNAs derived from UTRs of protein-coding genes. These genic piRNAs preferentially arise from 3′UTRs, and are produced by a piRNA biogenesis pathway that does not require ping-pong components, and are conserved across *Drosophila, *mice, and *Xenopus *(Robine et al., 2009). These breakthrough findings provide overwhelming evidence for an additional and much larger breadth of piRNA pathway mediated gene regulation, in addition to TGS of TEs, which still remains mostly unsolved.

The piRNA/Piwi pathway has several features that make it suitable as a mediator of epigenetic memory in germ cells. Mainly characterized by its ability to exert TGS by driving site-specific methylation of TE, the piRNA pathway clearly has the ability to impact genome stability in future generations. Moreover, even though this novel small RNA pathway has been shown to play a role in many of the epigenetic alterations that have been observed in response to IR, no experiments have been conducted to examine the possible role and response of this pathway to IR exposure. Because this pathway is mainly restricted to the male germline in mammals, it provides a novel mechanism to facilitate paternal epigenetic inheritance of IR-induced genomic instability. This could also provide some insight into the observed loss of LINE-1 and global DNA methylation, not only in the germline of exposed males, but more importantly, in the next generation (Koturbash et al., 2006a; Tamminga et al., 2008b; Filkowski et al., 2010). Understanding if and how the piRNA pathway responds to IR exposure could also potentially corroborate and help elucidate the increased mutation rates observed in satellite DNA and ESTR loci in the somatic and germline tissue from the progeny of exposed parents (Barber and Dubrova, 2006).

## Piwi, PiRNAs AND SPERMATOGENESIS

The mouse genome encodes three Piwi proteins, all of which play essential and non-redundant roles in virtually all stages of spermatogenesis (Deng and Lin, 2002; Kuramochi-Miyagawa et al., 2004, 2008; Carmell et al., 2007). Therefore, we will introduce the relevant stages and cellular associations of the rodent germline in order to further discuss, in context, the known roles of the piRNA pathway in spermatogenesis.

Starting from a self-renewing stem cell pool, male germ cells continually develop from puberty to old age/death. The complete process of male germ cell development is called spermatogenesis, and takes place within the testes (Holstein et al., 2003). A testis can be divide into several hundred (~370) lobules that consist of the seminiferous tubules and intertubular tissue. The intertubular tissue contains groups of endocrine Leydig cells, as well as additional cellular elements. The seminiferous tubules are coiled loops that are connected at both ends to the rete testis. The rete testis is a connecting network of delicate tubules located in the hilum of the testicle (mediastinum testis) that carries spermatozoa from the seminiferous tubules to the vasa efferentia. Fluid containing immature spermatozoa is secreted by the seminiferous tubules and collected in the rete testis to be delivered to the excurrent ductal system of the epididymis where the spermatozoa mature into functional sperm (Holstein et al., 2003).

**FIGURE 2:**
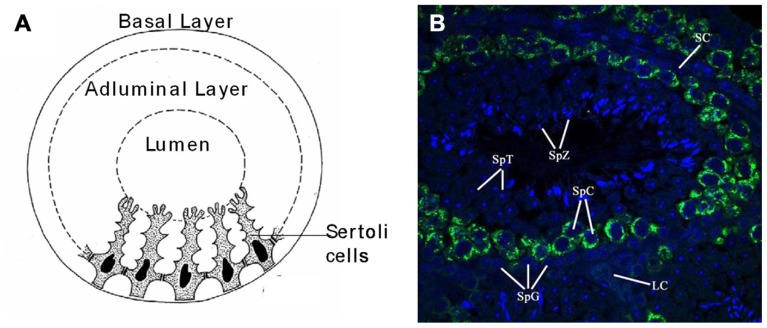
**Anatomy and cellular associations of a murine seminiferous tubule cross section.** **(A)** Drawing of a cross section of a mouse seminiferous tubule showing Sertoli cells dividing the germinal epithelium into basal and adluminal compartments. **(B)** Immuno-fluorescent picture of a seminiferous tubule from a cross section of paraffin embedded mouse testis with Mili (green) and nuclear (DAPI) stain (blue). Image taken with a laser scanning confocal microscope (×60). Seminiferous tubule labeled with relevant cell types associated with spermatogenesis: SpG, spermatogonia; SpC, spermatocyte; SpT, spermatid; SpZ, spermatozoa; SC, Sertoli cell; LC, Leydig cell.

The seminiferous tubules of the testes contain germ cells at various stages of development. The main stages of cell types, in sequential order of development, are known as spermatogonia, primary and secondary spermatocytes, and spermatids (**Figure 2B**). As the spermatogonia divide and mature into various cell types, they move progressively from the basal layer, through the adluminal compartment, to the lumen of the seminiferous tubule. As a germ cell progresses from the basal layer to the lumen of the tubule in what is known as a spermatogenic cycle, it passes through three major stages of development, which are referred to as spermatogoniogenesis, meiosis (of spermatocytes), and spermiogenesis (maturation of spermatids into spermatozoa; **Figures 2A, B**).

There are two types of spermatogonia, namely A-type and B-type. Type A spermatogonia belong to a self-renewing stem cell population, which divide continuously in successive mitosis to give rise to one A-type and one B-type spermatogonium. B-type spematogonia are committed to undergo further germ cell development, in which an additional mitotic division gives rise to two primary spermatocytes. This marks the end of spermatogoniogenesis and the beginning of meiosis. Cells in meiosis are called spermatocytes. As the process of meiosis comprises two divisions, cells before the first division are called primary spermatocytes (2*n*), and cells after the first meiotic division are referred to as secondary spermatocytes (1*n*). These secondary spermatocytes then undergo the second division of meiosis, giving rise to four haploid round spermatids. These immature spermatids differentiate into spermatozoa in a process called spermiogenesis. Spermiogenesis ends when these cells are released from the adluminal compartment of the germinal epithelium to the lumen, at which point the free cells are called spermatozoa. Importantly, these aforementioned germ cell divisions are usually incomplete. After germ cells divide, their daughter cells remain interconnected by cytoplasmic bridges so that a clone, derived from one stem cell, forms a syncytium of cells (Greenbaum et al., 2007). Syncytial connections are maintained through spermatogonial and spermatocytic stages, and are dissolved only in advanced phases of spermatid development. This allows for rapid communication between cells, and this is believed to be the basis for the synchronous development of germ cells (Greenbaum et al., 2007). The aforementioned germ cells of the seminiferous epithelium are located within invaginations of somatic Sertoli cells. These Sertoli cells are connected by specialized zones of tight junctions that separate the germinal epithelium in basal and adluminal compartments (**Figure 2A**). These specialized zones, or so-called “tight junctions,” form the blood–testis barrier (Parreira et al., 2002). Once maturing germ cells pass this blood–testes barrier, they are protected from exogenous substances, as well as the host’s immune system (Itoh et al., 2005). Sertoli cells also function as “nurse cells” that regulate the flow of nutrients and growth factors required by germ cells (Petersen and Soder, 2006). Furthermore, Sertoli cells are also involved in the production of endocrine and paracrine substances that regulate spermatogenesis and the movement of germ cells within the seminiferous epithelium (Mruk and Cheng, 2004; Petersen and Soder, 2006).

Normally, a new cycle of spermatogenesis begins before the preceding cycle has finished and depending on the length of spermatogenesis and the frequency of new cycles a cross section of the testis should reveal several hundred seminiferous tubules, each having a particular cellular association (Hess et al., 1990). These particular cellular associations have been categorized into a number of stages that make up a spermatogenic cycle, with 12 and 14 specific stages being identified in mice and rats, respectively (Hermo et al., 2010). The seminiferous tubules are organized in such a way that these stages occur in a consecutive order. The sequential order and repetition of each stage along the tubule produces what is known as a “spermatogenesis wave” (Hermo et al., 2010).

Three murine Piwi-like proteins, Miwi (Piwil1), Mili (Piwil2), and Miwi2 (Piwil4), are essential and required for different stages of spermatogenesis. Moreover, they bind to distinct classes of their piRNA partners which are expressed during spermatogenic cycles, with particular sequence content distinguishing piRNA populations from embryonic and pre-meiotic germ cells from those that appear during meiosis throughout spermatogenesis (Aravin and Hannon, 2008).

The expression of Miwi begins shortly after birth (14 dpp) and continues until old age/death starting in the pachytene stage of meiosis (spermatocytes) and into the round spermatid stage of germ cells during spermatogenesis (Deng and Lin, 2002). *Miwi*-null spermatocytes will arrest post-meiotically at the round spermatid stage (Deng and Lin, 2002), although the basis for this developmental defect is unknown, Miwi has been posited to act in translational control, and loss of this control is thought to be a contributing factor (Grivna et al., 2006). Furthermore, the expression of Miwi strongly coincides with spermiogenesis, when chromatin is packed in such a manner that transcription does not occur at a significant level (Yu et al., 2003), at which point cells rely on stored mRNAs and post-transcriptional control of gene expression (Penttila et al., 1995; Yang et al., 2005b). During meiosis Miwi and Mili have overlapping expression patterns, during which time they both interact with an extremely abundant class of small piRNAs, known as pachytene piRNAs, the function of which remains elusive (Aravin et al., 2006; Girard et al., 2006). This class of pachytene piRNAs, derived mainly from non-repetitive genomic regions is, for the most part, lost in *Miwi *mutants, which is also thought to be partially responsible for the post-meiotic arrest of spermatogenesis in these animals (Aravin et al., 2006; Girard et al., 2006).

Of the three murine Piwi proteins, Mili is the most broadly expressed. Mili is detected in primordial germ cells (PGS) at 12.5 dpc, and persists during spermatogenesis up until the round spermatid stage (Aravin et al., 2008). Mili not only has overlapping temporal expression with both Miwi and Miwi2, but also associates with all developmental stage-dependent classes of piRNAs (i.e., prenatal/prepachytene, and pachytene piRNAs; Aravin et al., 2007b, 2008; Kuramochi-Miyagawa et al., 2008).

The expression pattern of the third murine Piwi protein, Miwi2, is the most restricted, seen only perinatally in germ cells (gonocytes) from 15.5 dpc until a few days after birth (Aravin et al., 2008). *Mili *and *Miwi2 *mutants show quite similar phenotypes with the arrest of germ cell development due to apoptosis at the early pachytene stage of meiosis (Kuramochi-Miyagawa et al., 2004; Carmell et al., 2007). Both mutants also exhibit enhanced retrotransposon expression in the male germline due to defective *de novo *DNA methylation of the derepressed TEs (Kuramochi-Miyagawa et al., 2008). The time of overlapping expression of Mili and Miwi2 also coincides with the critical window of time during which male gametic *de nov*o methylation patterns are established (Lees-Murdock et al., 2003; Kato et al., 2007). It is now accepted that Mili and Miwi2 play distinct but complementary roles in establishing *de novo *methylation patterns that silence TEs in developing male germ cells. This was originally discovered because of their interactions with a discrete population of piRNAs (prepachytene/prenatal) that are expressed at this time (Aravin et al., 2008). These piRNAs are primarily derived from repetitive genomic regions, and show features of a “ping-pong” amplification cycle that drives the sequence-specific methylation of TEs, while selectively consuming active TE transcripts to drive the generation of new piRNAs (Aravin and Bourc’his, 2008; Aravin and Hannon, 2008; Aravin et al., 2008; **Figure 1**). Before describing the ping-pong amplification cycle, it is necessary to define primary and secondary piRNAs. In general, piRNAs are designated as primary, not necessarily because of their order of production, but because they have a strong preference for a 5′ uridine (1U). Pachytene piRNAs are exclusively primary; however, there is a subset of prepachytene piRNAs that are generated in the ping-pong cycle which are defined as secondary piRNAs and characterized by an adenine 10 nt from the 5′ end (10A) (Aravin et al., 2008; **Figure 1**).

In the mammalian ping-pong cycle, it is believed that sense transcripts, likely mRNAs of active transposons, represent the major substrate for primary processing (process unknown) of piRNAs that then associate with Mili (Aravin et al., 2008; **Figure 1**). These primary sense piRNAs then guide Mili toward recognizing and cleaving antisense transcripts (possibly transcribed from genomic piRNA gene clusters) that contain transposon sequences (**Figure 1**). This produces a secondary antisense piRNA that complexes with Miwi2 (**Figure 1**). Miwi2 and its secondary antisense piRNA partner can then either continue in this ping-pong cycle by recognizing complementary RNA transcripts (e.g., transposon mRNA), essentially regenerating a primary sense piRNA that would associate with Mili, or it can guide sequence-specific DNA methylation of TE in the nucleus (Aravin et al., 2008; **Figure 1**). Genetic and molecular characterizations of the interactions between methyltransferases and the piRNA pathway are consistent with piRNA/Piwi complexes directing DNMT3L, and indirectly active methyltransferases (DNMT3a, DNMT3b), to target loci based upon the sequence of their bound, small RNA guides (Aravin and Bourc’his, 2008; Aravin and Hannon, 2008; Aravin et al., 2008).

## DISCUSSION/CONCLUSION

Owing to a marked increase in accessibility and the introduction of innovative techniques that utilize IR, the number of people that receive exposure to radiation via occupational, diagnostic, or treatment-related modalities is progressively rising. The problem of potentially heritable deleterious effects associated with radiation-exposed parents has become an issue of utmost importance. A major quality of life issue faced by young people, who are exposed to radiation, is not only an increased risk of secondary cancer development, but also the ability to produce healthy offspring. The primary negative biological effects of IR have historically been accepted as direct damage to DNA. It is now known that this damage, in conjunction with the disruption of a variety of cellular regulation processes, can lead to the phenomenon of genomic instability that is linked to carcinogenesis (Little, 2000; Barcellos-Hoff, 2005; Sowa et al., 2006). IR-induced genome instability can occur in the descendents of directly exposed cells for many generations, as well as in naïve un-irradiated bystander cells (Morgan, 2003a, b).

The testes are one of the most radiosensitive organs (Feinendegen, 2005), and could play a key role in facilitating transgenerational genomic instability. Even if IR exposure is directed to distant body parts, it can lead to genomic instability in the germline and further to transgenerational genome instability in the unexposed offspring of parents exposed before conception (Dubrova, 2003b; Morgan, 2003a, b, c; Tamminga et al., 2008b). Although it is clear that IR-induced bystander and transgenerational effects are linked to genome instability, the exact molecular mechanisms that lead to their development are only beginning to be understood. Accumulating evidence suggests that epigenetic alterations are key factors underling the molecular etiology of transgenerational effects such as genome instability (Kovalchuk and Baulch, 2008).

DNA methylation is one of the main epigenetic mechanisms that safeguard genome stability in cells, including regulating gene expression and chromatin structure. The germline-specific piRNA pathway has an established role in maintaining genome stability as it enforces the silencing of transposable elements by directing site-specific methylation during male germ cell development (Aravin et al., 2007a, b; Aravin and Hannon, 2008; Kuramochi-Miyagawa et al., 2008). As such, the piRNA pathways represent perhaps the only currently known sequence-specific mechanism for deposition of DNA methylation in mammals. It remains to be seen if IR exposure induces a piRNA/PIWI pathway response, and if these responses are involved in the molecular and epigenetic consequences associated with direct and indirect radiation exposure upon the male germline. The piRNA pathway does provide a novel epigenetic mechanism poised to be involved in transgenerational radiation effects, such as genome and epigenomic instability. In addition to being directly involved in the maintenance of genomic instability, by facilitating DNA methylation of TE, the piRNA pathway has been implicated in the other epigenetic alterations that affect a variety of cellular regulation processes. However, no studies have ever been undertaken in order to examine whether the piRNA/Piwi pathway plays a role in germline responses to radiation exposure. Further studies are clearly needed to understand the possible molecular, biological, and evolutionary consequences of piRNA pathway changes that may be induced by radiation exposure and the impact this may have on male germline genome integrity.

We think that the piRNA/Piwi pathway, necessary for epigenetic regulation of genome stability in the male germline, may play a role in the epigenetic alterations involved in the production/inheritance of IR-induced genomic instability.

Furthermore, we predict that the piRNA/Piwi pathway plays a role in the epigenetic inheritance of radiation-induced genomic instability. Future studies will be required to examine the effect of IR exposure on piRNA/Piwi pathway components. Analysis of Piwi proteins and sequencing of their piRNA partners will direct us in understanding the effects of IR-induced alterations to the piRNA/Piwi pathway on the epigenome. With the rate of advancement in sequencing techniques and bioinformatics we will soon be able to identify the functional targets of piRNAs, which will guide us in understanding the biological consequences of piRNA pathway responses to IR that may be linked to heritable effects associated with IR exposure (i.e., transgenerational genomic instability and carcinogenesis).

## Conflict of Interest Statement

The authors declare that the research was conducted in the absence of any commercial or financial relationships that could be construed as a potential conflict of interest.
